# Artificial intelligence approaches for schizophrenia prediction and its biomarkers using medical imaging data

**DOI:** 10.3389/fpsyt.2026.1821091

**Published:** 2026-05-12

**Authors:** Suresh Babu Palpandi, Nagaraj Palanigurupackiam, Hessa Almatar, Reema Alduhayan, Barrak Alsomaie, Ahmed Almazroa

**Affiliations:** 1 Department of Computer Science and Engineering, School of Computing, Kalasalingam Academy of Research and Education, Krishnankoil, Tamil Nadu, India; 2 Department of Computer Science and Engineering, School of Computing, SRM Institute of Science and Technology, Tiruchirappalli, Tamil Nadu, India; 3 AI and Data Management, King Abdullah International Medical Research Center, Riyadh, Saudi Arabia; 4 King Saud bin Abdulaziz University for Health Sciences (KSAU-HS), Riyadh, Saudi Arabia; 5 Research Operation Department, King Abdullah International Medical Research Center, Riyadh, Saudi Arabia

**Keywords:** artificial intelligence, biomarker, deep learning, machine learning, multimodal data, neuroimaging, schizophrenia, unimodal data

## Abstract

**Background:**

Schizophrenia (SZ) is a debilitating mental illness that adversely affects social and family interactions, ranking as a leading contributor to global disability. Existing diagnostic approaches, including MRI, PET, and EEG, underscore the necessity for effective predictive strategies to enhance management and reduce costs.

**Objective:**

This review evaluates the application of artificial intelligence (AI) methodologies—specifically Machine Learning (ML) and Deep Learning (DL) in predicting SZ using medical imaging data, while addressing existing challenges and identifying key biomarkers to improve diagnostic accuracy.

**Methods:**

A systematic literature review was performed using the databases IEEE, PubMed, ScienceDirect, MDPI, Google Scholar, and Springer from inception until March 31, 2026. The initial search generated 820 records, and after a thorough screening process, 185 studies relevant to disease diagnosis, model selection across various neuroimaging modalities, including biomarker identification, were identified. The review protocol has been registered with PROSPORO registration: CRD420251131635. The studies were selected based on different medical imaging data related to SZ.

**Results:**

This review presents a thorough examination of advancements in SZ detection via AI methodologies. It highlights not only providing existing predictive techniques, identifies research gaps, biomarkers identification and assessment, and underscores the potential of AI-based ML and DL methods to facilitate early and accurate diagnosis of SZ. Five unimodal and various combinations of multimodal data were examined, along with the AI models’ performance metrics from multiple studies.

**Conclusions:**

The review provides a comprehensive assessment of AI algorithms relevant to both unimodal and multimodal data, biomarkers of neuroimaging modalities with ROIs, challenges, and limitations of ML and DL models, and future directions of prediction for clinical diagnosis, thereby supporting timely interventions for individuals affected by SZ.

**Systematic review registration:**

https://www.crd.york.ac.uk/PROSPERO/view, identifier CRD420251131635.

## Introduction

SZ is a debilitating and severe mental illness that poses a high risk to affected individuals (1). This condition significantly impacts patients’ behavior in social and familial contexts and is among the top ten global causes of disability, according to the World Health Organization (2). Approximately 3% of the worldwide population is affected by bipolar disorder or SZ (3). Individuals diagnosed with SZ have a higher mortality rate compared to the general population, with suicide being a leading cause of death among these patients (3–5).

Symptoms of SZ often manifest between the ages of sixteen and thirty, following an initial phase of psychosis (6). These symptoms can be categorized into positive symptoms (such as hallucinations), negative symptoms (like diminished emotional expression), and cognitive symptoms (such as trouble focusing)^6^. Additionally, patients experience structural brain changes, including the enlargement of the lateral and third ventricles and reductions in the volumes of critical brain areas (7).

Current diagnostic methods primarily rely on MRI (Magnetic Resonance Imaging), PET (Positron Emission Tomography), and EEG (electroencephalogram) scans, alongside subjective assessments of patients’ behaviors and expressions. The annual financial burden associated with SZ ranges from $94 million to $102 billion (7), placing a significant strain on individuals, families, and society as a whole (8). Therefore, effective disease prediction is crucial for managing the illness and controlling costs.

Recent advancements in AI, particularly in ML and DL, have enabled systems to identify patterns in data (9, 10) autonomously. These approaches offer benefits such as improved disease recognition, reduced healthcare costs, and minimized medical decision-making errors (11). While various studies have explored the use of AI in healthcare, there remains a gap in comprehensive reviews specifically addressing AI approaches for predicting SZ using medical imaging data.

The review article of Lai et al. offers a detailed investigation of the application of AI and ML algorithms in the diagnosis and classification of schizophrenia. The article describes the utilization of diverse methods such as support vector machine (SVM), KNN, decision tree analysis, and deep learning. Different data types were analyzed, such as those obtained with the help of MRI, EEG, and clinical tests. The authors emphasize the fact that AI technologies are capable of extracting discriminant characteristics from complicated and high-dimensional data, which makes diagnosis more accurate than that provided by conventional clinical assessment methods. The article further outlines a typical ML process that includes feature extraction, feature selection, model training, and validation. The authors also identify some challenges faced in clinical applications of the reviewed methods (12).

This review article by Sun Hui et al. covers the current utility of MRI in clinical practice for schizophrenia patients, with an emphasis on the use of MRI technology in diagnosing, prognosing, and assessing treatments of the disease. Consistent findings using MRI include both morphological changes (such as reduced gray matter) and functional or connectivity changes in the brain, all reflecting underlying pathophysiology associated with schizophrenia. In addition, MRI is shown to have potential in identifying high-risk individuals, predicting disease progression, as well as the effectiveness of certain treatments. One interesting finding from the paper includes the increasing use of machine learning and deep learning algorithms to analyze MRI results, providing more personalized predictions of diagnostic and prognostic information. One major drawback mentioned in this paper is the discrepancy between current research findings and their practical implementation (13).

This review aims to provide a thorough investigation of methodologies, biomarkers, challenges & limitations to ML and DL models, and findings related to the use of AI in predicting SZ through medical imaging with clinical perspective. It will explore both ML and DL techniques while addressing challenges related to data adequacy, quality, interpretability, clinical context, and ethical considerations. It provides the detailed information about biomarkers of SZ and its assessment, challenges when considering its prediction. The study details how SZ diagnoses are conducted using advanced learning models, covering diverse data types, preprocessing techniques, features, biomarkers, and future research directions. Identifying biomarkers is crucial for recognizing individual traits, leading to more precise medical interventions and improved outcomes.

The remainder of this paper is organized as follows: Section 2 interprets the review methodologies according to PRISMA guidelines. Section 3 examines the classification and detection of SZ using various data modalities. Section 4 reveals the biomarkers and their related Regions of Interest related to neuroimaging data associated with SZ. Sections 5 and 6 summarize predictions of SZ using ML and DL models, respectively. Section 7 outlines the challenges and limitations of these models, while Section 8 concludes with key considerations regarding the detection of SZ through ML and DL approaches.

## Methods

### Design

**Figure 1:**
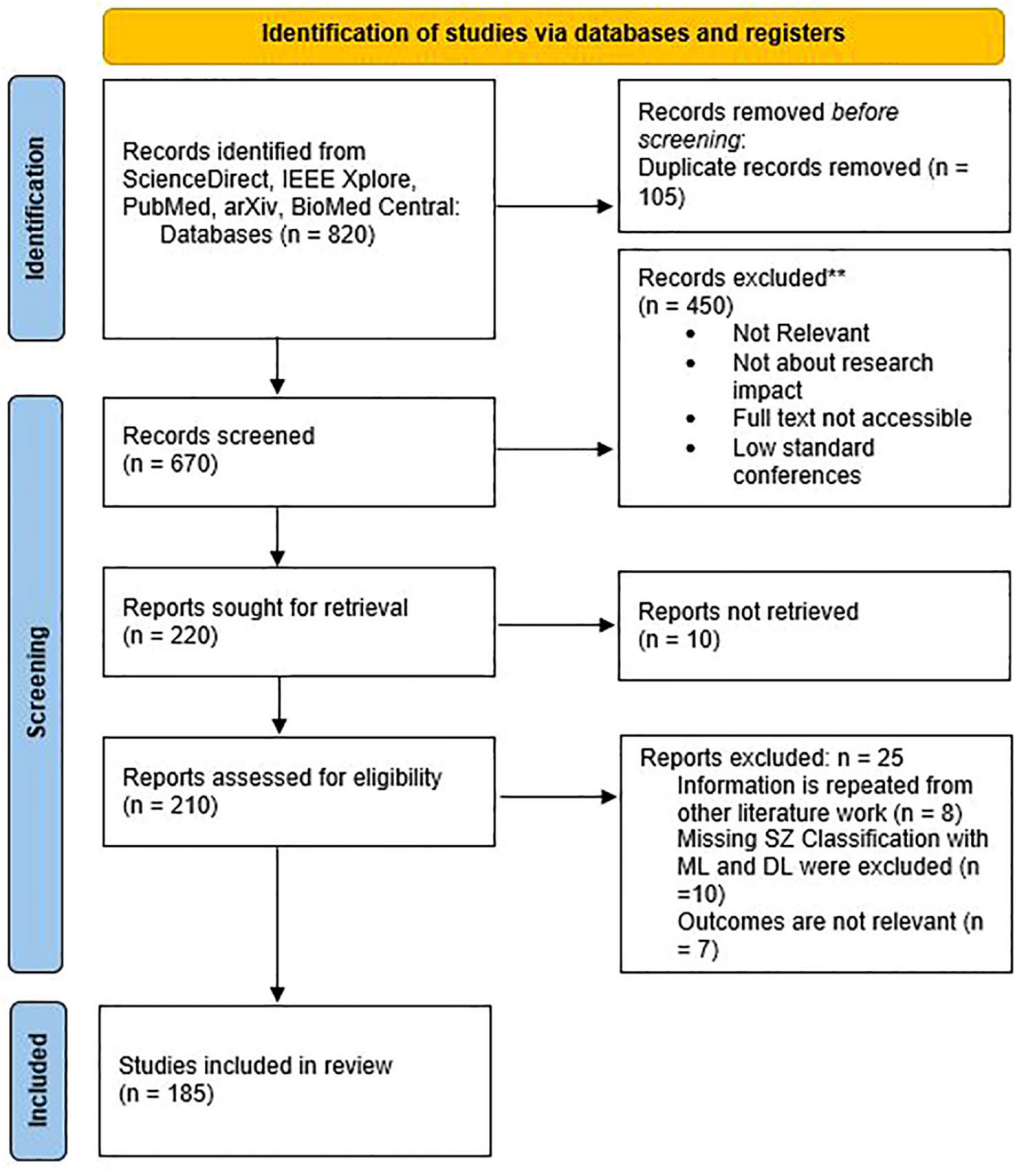
Procedural flowchart following the PRISMA guidelines.

The Preferred Reporting Items performed a systematic review of the literature for Systematic Reviews and Meta-Analyses (PRISMA) guidelines. The PRISMA guidelines ensure the highest quality in scientific research. To more fully present the results of the review, a narrative form was additionally adopted. The electronic databases PubMed, Web of Science, IEEE, ScienceDirect, and MDPI were searched from January 1999 to March 2026. A keyword search using the Boolean operators OR & AND with terms including “schizophrenia,” “diagnosis,” “artificial intelligence,” “mental health,” “Machine Learning,” “Deep Learning,” “MRI,” “fMRI,” “sMRI,” “multimodal,” was conducted (Online Supplemental Data). Duplicates of search results from different databases were identified and removed. A PRISMA 2020 flow diagram is presented in **Figure 1**. The review protocol registered in the International Systematic Review Registry – PROSPERO (Prospective Register of Systematic Reviews) under registration number: CRD420251131635. The protocol used for this review is provided in the Online Supplemental Data. This review consolidates the selected papers by showcasing their sample sizes and the methods employed to assess the accuracy of these approaches. The PRISMA checklist is available in Multimedia Appendix 1.

## Results

### Classification and detection of SZ using different data modalities

Various AI and ML technologies have been employed to analyze MRI images for the identification of SZ. Standard MRI scans can help medical practitioners detect the onset of SZ (14).

#### Structural MRI

sMRI involves comparing MRI images from patients and healthy controls to assess brain function. Discriminant Functional Analysis (DFA) was notably applied by Leonard et al., achieving a 77% success rate in classifying individuals based on structural brain scans. Most studies focus on the densities and sizes of Grey Matter (GM) and White Matter (WM) (15).

Different modifications of DFA have been successfully utilized to categorize and diagnose SZ, often achieving improved prediction rates by incorporating various ROIs (15–17). Researchers consistently find that the likelihood of SZ is often determined by general brain deviance rather than specific structural anomalies.

Support vector machines (SVMs) are widely used for SZ classification, particularly non-linear SVMs, which have been effective in processing sMRI images (18). Koutsouleris et al. employed a non-linear SVM to classify sMRI data from patients with early and late At-Risk Mental State (ARMS) and Human Control (HC). Their approach achieved notable accuracy rates: 86% for HC, 91% for early ARMS, and 86% for late ARMS (18). Follow-up studies confirmed high accuracy in distinguishing between groups, with 90% accuracy for HC and 88% for those transitioning to psychosis.

Further research indicates that combining features from multiple modalities yields better prediction accuracies than single-modal approaches (19–21). Techniques such as Gradient Boosting (GB), Random Forest (RF), and Convolutional Neural Networks (CNN) have also been employed for improved results. Csernansky et al. used regression analysis to predict SZ, revealing significant morphological abnormalities in the hippocampus, though misclassifications highlighted the complexity of clinical features (22).

Davatzikos et al. demonstrated an average classification accuracy of 81.1% using multidimensional non-linear classification of patterns in sMRI data (23). Xiao et al. uses SVM classification on sMRI (thickness and surface area) of 163 drug-naïve FES patients compared to 163 controls; accuracy is 85% (surface area) and 82% (thickness). The high level of accuracy found in First-Episode SZ (FES) patients who have never been medicated shows that sMRI is useful for diagnosis (24).

Voxel Based Morphometry (VBM) study with 25 drug-naïve long-term SZ subjects compared to 33 healthy subjects revealed significant differences in neuroprogressive changes in the brain in an entirely drug-naïve chronic SZ patient. Subjects diagnosed with SZ had thinner cortex in the bilateral ventromedial prefrontal cortices, left superior temporal gyrus, and right pars triangularis than their counterparts, along with thicker cortex in the left superior parietal lobe. This research proves that neuroprogression occurs naturally as a result of the disease irrespective of any medication, and prefrontal-temporal thinning is a critical biomarker of neuroprogression in SZ (25).

Zhang et al. intends to build a first prototype of an efficient and practical tool to screen individuals at risk of mental illness. Specifically, Zhang et al. builds an advanced model, which is a Multiple Instance Learning DL Model, based on routine clinical MRI images collected from 14,915 severe mental illness (SMI) patients (SZ, bipolar, major depression) versus 4,538 normal people with high sensitivity (0.77). This study employed the largest sample size ever reported, consisting of clinical MRI images of several common types of SMI for the purpose of discriminating SMI patients by means of a machine learning technique. The advanced deep learning model constructed based on the use of clinical MRI images for the purpose of automatically detecting patients with SMI showed promising results in comparison to other models (26).

In yet another study, a multi-task model of deep learning was created based on raw anatomical MRI data collected from 286 first episode drug-naïve schizophrenic patients along with 330 healthy controls by including classification and regression tasks that classify patients and control subjects and measure the severity of symptoms in terms of PANSS score; accuracy achieved was 83%, higher than single task and baseline models (27). An overview of AI models predicting SZ using sMRI is presented in **Table 1**, summarizing sample sizes and model performance.

**Table 1:** A summary of different AI and ML models to predict SZ using sMRI scans.

Study	Year	Subjects		Prediction	AI technique
		Patients	Control		
Fan et al. (32)	2006	69	79	91.8%♀, 90.8%♂	Nonlinear SVM, leave-one-out cross-validation
Yoon et al. (33)	2007	53	52	at least 88.8%	SVM, Principal Component Analysis (PCA)
Kawasaki et al. (17)	2007	46	46	90%, 80%, 75% (Jackknife)	Multivariate Linear DFA, Jackknife approach
Pohl and Sabuncu (34)	2009	16	17 (age-matched)	up to 90%	Linear SVM, Leave-one-out cross-validation
Koutsouleris et al. (35)	2009	A1: 20 (ARMS-E), 25 (ARMS-L) A2: 15 (ARMS-T), 18 (ARMS-NT)	A1: 25 (matched) A2: 17 (matched) Cross-validation: 45	at least 86% (sensitivity) at least 93% (specificity)	SVM, Multi-variate Pattern Analysis (MVPA)
Castellani et al. (36)	2010	64	60	up to 86.13%	SVM
Koutsouleris et al. (18)	2010	25	28	83%	SVM with Partial Least Squares Pattern Analysis
Koutsouleris et al. (37)	2012	16(ARMS-T)/21(ARMS-NT)	22	ARMS – T Vs HC: 92.3% ARMS – NT Vs HC: 66.9% ARMS –T Vs ARMS –NT: 84.2%	SVM
Ulaş et al. (19)	2012	50	50	84.0% (MKL) 77.0% (SVM)	SVM, Multiple Kernel Learning (MKL)
Ulaş et al. (20)	2012	42	40	90.24% (CLMKL) 71.95% (SVM)	SVM, Clustered Localized MKL (CLMKL)
Bansal et al. (38)	2012	65	40	93.1% (sensitivity) 94.5% (specificity)	Hierarchical clustering, Split-half, and Leave-one-out cross-validation
Perina et al. (39)	2014	42	40	83% (sensitivity)	SVM
Schnack et al. (40)	2014	46 SZ/47 bipolar disorder (BP)	43	SZ Vs HC: 90.0% SZ Vs BP: 88%	SVM
Lu et al. (41)	2016	41	42 (sex- and age-matched)	91.9% (sensitivity) 84.4% (specificity)	SVM, Recursive Feature Elimination (RFE)
Chin et al. (42)	2018	141	71	92%	Regularized SVM
Xiao et al. (24)	2019	163 drug-naïve FES	163	85% on surface area, 82% on cortical thickness	SVM
Chen et al (43).	2020	34	34	85%	Linear SVM
Tanveer et al. (44)	2022	72	74	80.71%	SVM and RF
Hu et al. (45)	2020	289	210	79.27%	3D CNN
Oh et al. (46)	2020	424	449	97%	3D CNN
Li et al. (47)	2021	89	83	99.72%	CNN
Hu et al. (48)	2022	289	210	79.27%	CNN
Wen et al. (49)	2022	38	39	74.9%	3D CNN
Zhang et al. (50)	2023	437	450	92%	3D CNN
Cui et al. (51)	2022	662	613	85.74%	Deep Neural Network (DNN)
Zhang et al. (26)	2023	14,915 patients with SMI, 148 Real-world high stress Mental Illness	4538	82%	Multiple Instance Learning (MIL)
Zhang et al. (25)	2015	25	33	p < 0.01	Pure statistical method
Zhang et al. (27)	2025	286 drug-naïve FES	330	83%	3D CNN

#### Functional MRI

fMRI studies examine brain metabolism through changes in blood oxygen levels, making it a valuable tool due to its higher spatial resolution. Recent advancements have improved the signal-to-noise ratio, allowing for more complex analyses. Various statistical methods, including SVM, Independent Component Analysis (ICA), and PCA, are used to analyze fMRI data.

Notable studies by Calhoun et al. (28) and Jafri and Calhoun (29) revealed that SZ patients activate fewer brain regions during cognitive tasks compared to controls. This finding has led to increased interest in using fMRI for clinical investigations of SZ. Their groundbreaking work demonstrated a mean accuracy of 75.6% in estimating functional brain modes from resting state data, with subsequent studies achieving sensitivities of 92% and specificities of 95% using multivariate analytical methods (30).

Another RS-fMRI study (ReHo approach) involving comparison between subjects with schizophrenia who had never been treated (Non-Treated-SZ, n=33), subjects with schizophrenia being under treatment (Treated-SZ, n=41), and healthy subjects (HC, n=74). Results of this RS-fMRI investigation (ReHo – Regional Homogeneity) showed that functional disorganization of white matter is the major dysconnection pattern in chronic patients with schizophrenia regardless of their treatment status. Abnormal functioning and connectivity of the splenium of corpus callosum appeared higher in subjects with untreated schizophrenia than those under treatment in comparison to healthy subjects (31). An overview of ML models using fMRI is illustrated in **Table 2**.

**Table 2:** An outline of the work, sample size, and accuracy that came from using ML on fMRI data.

Study	Year	Subjects		Prediction	AI/ML technique
		Patients	Control		
Jafri and Calhoun (29)	2006	38	31	75.6%	Neural network
Arribas et al. (52)	2010	21	25	90%	Kullback-Leibler divergence reduction for stochastic gradient learning.
Yang et al. (53)	2010	20	20	at least 82% (using fMRI data)	SVM
Castro et al. (54)	2010	52	54	95%	Different types of kernels, linear and Gaussian SVM, and leave-two-out cross-validation
Costafreda et al. (55)	2011	32	40	92% (Sensitivity)	SVM
Fan et al. (56)	2011	31	31	up to 85.5%	Linear kernel, Sigmoid kernel, radial basis function kernel, and SVM
Du et al. (57)	2012	28	28	90%	Linear discriminant analysis by Fisher, Network mode by default, Cross-validation with a majority vote, and leave-one-out
Liu et al. (58)	2012	25	25 (siblings) 25 (HC)	80.4% (*SZ* vs. HC)	Nonlinear SVM with polynomial kernel
Venkataraman et al. (59)	2012	18	18	75%	Multivariate classification
Fekete et al. (60)	2013	8♂	10♂	88%	Complex network analysis, Block diagonal optimization.
Yu et al. (61)	2013	32 (*SZ*) 19 (Depression)	38	80.9%	SVM, Intrinsic Discriminant Analysis, Leave-one-out cross-validation
Brodersen et al. (62)	2014	41	42	78%, 71%	Linear SVM, Variational Bayesian Gaussian mixture
Castro et al. (63)	2014	31	21	90% (L-norm MKL), 85% (Lp-norm MKL)	L-norm and Lp-norm MKL
Guo et al. (64)	2014	69	62	68%	SVM
Watanabe et al. (65)	2014	54	67	at least 77.0%	Fused Lasso and GraphNet regularized SVM
Cheng et al. (66)	2015	415	405	73.53–80.92%	SVM
Chyzhyk et al. (67)	2015	40	28	97–100%	Linear SVM
Peters et al. (68)	2016	18	18	up to 91%	SVM, Leave-one-out cross-validation
Yang et al. (21)	2016	40	40	77.91%	Maximum-uncertainty Linear Discriminant Analysis (MLDA), SVM
Skåtun et al. (69)	2017	182	348	up to 80%	Multivariate regularized LDA
Chen et al. (70)	2017	20 (*SZ*) 20 (depression)	20	60% (sensitivity) 90% (specificity)	Linear SVM, MVPA
Guo et al. (71)	2017	28	28 family-based control (FBC) 40 (HC)	SVM: 96.43% (sensitivity) 89.29% (specificity, FBC)	SVM, ROC curve
Iwabuchi and Palaniyappan (72)	2017	71	62	80.32%	MKL
Bae et al. (73)	2018	21	54	92.1% (SVM)	Various (5 types), 10-fold cross-validation
Li et al. (74)	2019	60	71	76.34% (LDA)	K-Nearest Neighbors (KNN), Liner SVM, Radial basis SVM, LDA
Chatterjee et al. (75)	2019	34	34	94% (SVM) 96% (1-NN)	SVM, k-nearest neighbors
Kalmady et al. (76)	2019	81	93 (sex- and age-matched)	87%	L2-regularized Logistic regression
Algumaei et al. (77)	2022	70	70	98.57%	SVM
Liu et al. (78)	2019	28	28	92.9%	SVM
Nimkar et al. (79)	2018	42	53	94.12%	SVM, KNN, RF and LDA
Han et al. (80)	2017	39	31	79.3%	Feed Forward Back Propagation Neural Network
Zeng et al. (81)	2018	474	607	85%	Discriminant Autoencoder Network with Sparsity constraint (DANS)
Oh et al. (82)	2019	103	41	84.43%	3D CNN
Yan et al. (83)	2019	558	542	83.2%	Recurrent Neural Network (RNN)
Zhao et al. (84)	2021	558	542	85%	Convolutional RNN
Smucny et al. (85)	2021	139	138	70%	Multilayer Perceptron (MLP)
Wang et al. (86)	2020	60	71	82.4%	Multi-Kernel Capsule Network (MKCN)
Yang et al. (31)	2020	33 Non-Treated (NT) SZ + 41 Chronic Treated (T)SZ	74	NT-SZ vs HC: 81.2% T-SZ vs HC:89.7%	SVM
Liu et al. (13)	2025	56	56	92.41%	SVM

#### Perfusion MRI and diffusion MRI

Emerging evidence suggests that cognitive impairments in SZ are linked to connectivity issues between brain regions rather than localized deficits. Techniques like dMRI and Diffusion Tensor Imaging (DTI) visualize water flow in the brain, providing insights into WM connectivity (87, 88).

pMRI assesses parameters such as cerebral blood flow and volume, offering valuable insights into SZ and related conditions (89, 90). These techniques have proven useful in evaluating treatment efficacy. A summary of studies applying ML to DTI and pMRI data is presented in **Table 3**.

**Table 3:** Utilizing a variety of AI methods and machine learning algorithms, this summary details previous work for the identification of SZ utilizing data from diffusion-weighted MRI, DTI, and pMRI images.

Study	Year	Subjects		Prediction	AI/ML technique
		Patients	Control		
Caan et al. (91)	2006	34	24	(not reported)	LDA, PCA
Caprihan et al. (92)	2008	45	45 (age-matched)	(not reported)	Discriminant PCA (DPCA)
Ingalhalikar et al. (93)	2010	27	37	90.62%	Nonlinear SVM
Rathi et al. (94)	2010	21 First Episodic Psychosis (FEP)	20 (age-matched)	SH: 78% (sensitivity) 80% (specificity) F2T: 86% (sensitivity) 85% (specificity)	KNN, Parzen window classifier, SVM
Ardekani et al. (95)	2011	50	50 (age- and sex-matched)	Based on FA: 94% accuracy, 96% sensitivity, 92% specificity Based on Mean Diffusivity (MD): 98% accuracy, 96% sensitivity, 100% specificity	Fisher’s LDA
Squarcina et al. (96)	2015	35 (FEP)	35	83%	SVM
Sutcubasi et al. (97)	2019	39	23	81.25%	Artificial Neural Networks (ANN)

#### PET scan

Positron Emission Tomography (PET) scans utilize radioactive tracers to visualize areas of increased metabolic activity in the brain. Studies indicate a role for neuroinflammation in SZ, with microglia being significant contributors. Levy et al. (98) found distinct cortical patterns in SZ patients, with high classification rates for visual tasks.

Josin and Liddle (99) conducted a neural network investigation to identify differences in functional connectivity patterns between 16 SZ patients and 6 HC. The neural network accurately classified all individuals in a test set comprising four healthy volunteers and nine SZ patients after training on data from two healthy individuals and seven SZ patients.

Bose et al. (100) differentiated 31 HC from 19 SZ patients using o-dihydroxyphenylalanine (DOPA) rate constants in the striatum, achieving 89% sensitivity and 94% specificity with an artificial neural network model. Despite relatively high classification accuracy, the limited sample size raises concerns about the reliability of PET scans as a standalone method for identifying SZ. However, the limited sample sizes in PET studies raise concerns about their reliability in SZ identification.

#### Composite data for classification and detection

The debate surrounding the most effective data types for medical AI predictions, especially in mental health, continues. Several studies have assessed the effectiveness of various ML approaches across different imaging modalities (53, 101–104). While many investigations have focused on single data types, the combination of modalities has shown promise.

In SZ multimodal prediction, the main ways to combine features are early fusion, late fusion, and intermediate/hybrid fusion. The choice depends on whether you want to merge raw features first, combine model outputs later, or learn a shared representation across modalities. Multimodal SZ prediction studies typically combine features using early fusion, late fusion, or intermediate fusion. Classical neuroimaging studies often use multivariate methods, whereas DL studies increasingly use attention-based fusion layers, concatenation, or ensemble voting. These strategies aim to exploit complementary information across modalities and improve classification performance over single-modality models (105, 106).

For instance, Hu et al. utilized SVM to analyze multimodal data, achieving 80% specificity and 75% sensitivity in distinguishing SZ cases (107). Similarly, Pettersson-Yeo et al. integrated genetic, DTI, sMRI, and fMRI data, demonstrating improved classification accuracy (108).

Research by Rahaman et al. employed unimodal and multimodal fusion, achieving accuracies of up to 92% using mid-fusion with attention (109). Wang et al., The study involved brain imaging datasets and blood RNA sequencing datasets for 43 subjects with SZ and 60 age- and gender-matched healthy subjects, with multiple omics features being derived from brain morphology, brain connectivity at structural and functional levels, and gene transcription of SZ susceptibility genes. The multiscale data fusion technique used in this study and their results were found in relation to the neural network model, with a 16.57% improvement in the multimodal model relative to the average performance of the unimodal model (105).

The study by Zeng et al. considers the influence of anti-psychotic medication and duration of illness on the use of several MRI modalities (Cortical Thickness, Cortcial Surface Area, GM Volume, ReHo, and fALFF) in differentiating between patients of schizophrenia and controls through multi-kernel learning. Never-treated FES had moderate diagnostic power (73%), as did LTSZ (83%). However, both FES (94%) and LTSZ after treatment (98%) yielded excellent results, suggesting that anti-psychotic drugs significantly improve diagnostic power beyond that of illness alone. Duration of illness was less influential. Hippocampal GMV proved to be a consistent predictor, whereas other features were mostly associated with either untreated FES or treated LTSZ (110).

Yang et al. conducted a systematic review of 36 longitudinal MR studies, comprising 21 studies using structural imaging and 15 studies using functional imaging, investigating antipsychotic-induced changes in brain structure and function among first episode schizophrenia (FES) patients, which can either improve or complicate the use of biomarkers for therapeutic monitoring. The investigators summarized the changes in the treatment-responsive brain regions into potential biomarkers for prognostic purposes. Striatal Volume: One of the most sensitive treatment biomarker—rapid hypertrophy (correlation of r=0.65 with improvement in PANSS scores), but confounds the diagnosis of treated FES compared to controls. Hippocampal GMV: Most reliable disease biomarker—the least affected by early treatment. Frontotemporal Progression: Prognostic indicator for poor outcome—patients under high-dose and long-duration DUP exhibit twice the atrophy rates. Both anatomical and functional alterations in the brain following treatment were observed in the frontal and temporal lobes, basal ganglia, limbic system, and DMN (111).

**Table 4** summarizes various multimodal approaches for predicting SZ.

**Table 4:** Summary of study and predictions for the identification of SZ with multimodalities approaches.

Study	Year	Subjects		Modalities	Prediction	AI/ML technique
		Patients	Control			
Yang et al. (53)	2010	20	20	task-related fMRI (auditory oddball task) + Single Nucleotide Polymorphism (SNP) (genome data)	Accuracy 87%	SVM
Ulas et al. (124)	2011	59	55	sMRI + Diffusion Weighted Imaging (DWI)	Accuracy 85.96%	Support Vector Regression (SVR) Classifier
Du et al. (57)	2012	28	28	rs-fMRI + task-related fMRI (auditory oddball task)	Accuracy 98%	LDA
Ota et al. (102)	2013	25	25	sMRI + diffusion MRI	Accuracy 88%	LDA
Sui et al. (103)	2013	35	28	sMRI + rs-fMRI + diffusion MRI	Accuracy 79%	SVM
Guo et al. (125)	2024	140	205	sMRI + fMRI	Accuracy 89.86%	3D CNN + 2D CNN
Silva et al. (126)	2014	40	46	sMRI + fMRI	Accuracy 94%	Gaussian Process Classifier
Axelsen et al. (127)	2015	40	46	sMRI + fMRI	Accuracy 70%	Permutation
Cetin et al. (128)	2016	72	72	rs-fMRI + task-related fMRI (auditory oddball task) + MEG	Accuracy 90%	LDA, Naïve Bayes Classifier, SVM
Qureshi et al. (129)	2017	72	72	sMRI + fMRI	Accuracy 99.29%	Extreme Machine Learning (ELM), SVM, LDA, RF
Ebdrup et al. (130)	2018	45	58	EEG + DTI + sMRI + Cog	Accuracy 69%	Naïve Bayes, SVM, Decision Trees (DT), RF, AS (Auto-Sklearn), Logistic Regression
Lei et al. (131)	2019	295	452	sMRI + fMRI	Accuracy 90.83%	SVM
Gagana B (132)	2021	69	75	sMRI + fMRI	Accuracy 90%	AutoML
Masoudi et al. (133)	2021	64	81	sMRI + fMRI + DTI	Accuracy 99.35%	3D CNN
Masoudi et al. (134)	2022	64	81	fMRI + DTI	Accuracy 99.5%	Gated Recurrent Unit with 2D CNN, 3D CNN
Rahaman et al. (109)	2023	162	275	sMRI + fMRI + SNP	Accuracy 92%	ICA, Auto Encoder, Feed Forward Network (FFN) bidirectional Long-Short Term Memory (bi-LSTM)
Bi et al. (135)	2023	350	477	sMRI + fMRI	Accuracy 83.33%	ViT (Vision Transformer)
Shi et al. (136)	2021	71	74	sMRI + fMRI	Accuracy – 93.75%	LDA
Calhoun et al. (137)	2017	144	154	sMRI + fMRI	Alignment Score – 4.3	Feed Forward Neural Network
Srinivasagopalan et al. (138)	2019	69	75	sMRI + fMRI	Accuracy – 94.44%	DNN
Kanyal et al. (139)	2024	219	273	sMRI + fMRI + SNP	Accuracy 79.01%	DenseNet
Zeng et al. (110)	2022	179 FES Long-Term Ill SZ, 30 (LTSZ), 71 FES group after treatment, 93 long-term antipsychotic treatment	373	sRMI + fMRI	FES 94%, LTSZ 98%	MKL
Yang et al. (111)	2021	36	36	sMRI + fMRI	NA. Available Descriptive Analysis	purely mechanistic review
Wang et al. (105)	2024	43	60	sMRI + fMRI + DWI + blood RNA	Accuracy 71.43%	SVM, KNN, DT, RF

### Neuroimaging biomarkers

In recent years, some exciting discoveries have been made in the research on objective biomarkers of SZ, primarily focusing on genetic susceptibility genes, metabolic indicators, immune indices, brain imaging, electrophysiological characteristics, which used for the prediction and diagnosis of SZ. A Biomarker or Biological Marker is an objective measure that can be assessed through blood, cells, other bodily fluids, body parts functionalities, functional connectivity of body regions with or without working and tissues. This helps to understand the condition of the body and its transformation, diagnosing the diseases, predicting the disease stages and personalized treatment strategies and monitoring treatment progression.

To be useful, a biomarker must serve as a proxy for a clinically relevant metric and demonstrate adequate sensitivity, specificity, and predictive value. Neuroimaging is an excellent candidate for biomarker discovery in SZ, as it can detect phenotypic differences in illness targets at the molecular, cellular, and brain circuit levels. Mechanistically based biomarkers of this kind may provide direct measures of the pathophysiological underpinnings of the illness process.

Diagnostic biomarkers are developed to index biological processes associated with objective disease signatures, thereby aiding in the detection of disease stage. With advances in neuroimaging technology, neuroimaging has become a strong candidate as a diagnostic biomarker for SZ (45–47).

**Figure 2:**
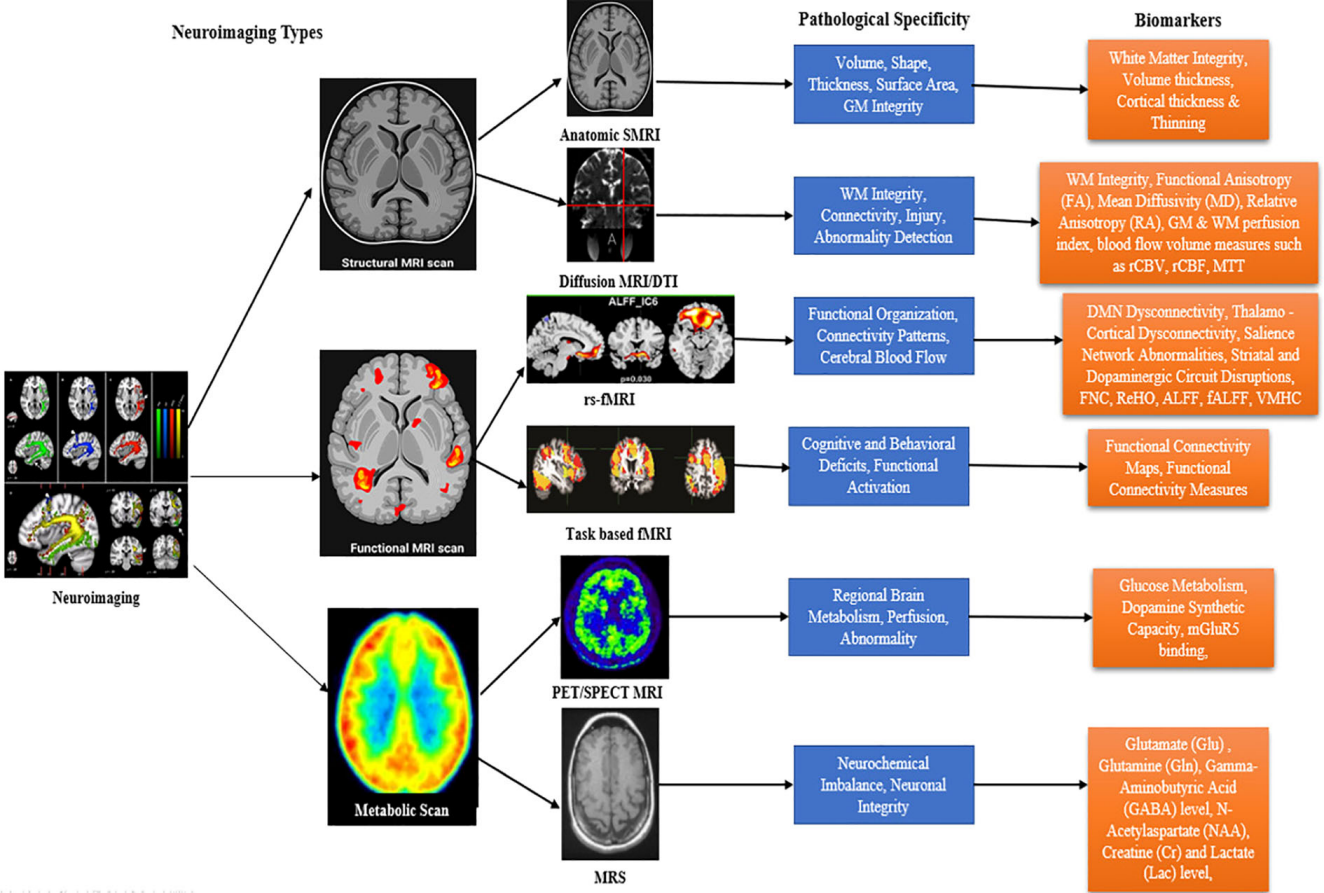
SZ neuroimaging biomarkers.

**Figure 2** presents biomarkers of SZ across different data modalities, and the following section provides a detailed interpretation of each neuroimaging modality’s biomarkers and their corresponding region of interest (ROIs).

#### sMRI

SZ showed reduced GM volumes in the hippocampus, amygdala, thalamus, nucleus accumbens and total intracranial volume, as well as larger pallidum and lateral ventricle volumes, especially in frontal and temporal lobe regions (17, 18, 24, 27, 32, 33, 35, 49–51, 79, 101, 112–123). The prefrontal, cingulate, and lateral temporal regions play important roles in working memory, executive function, and auditory sensation and language processing, all of which are impaired in SZ and psychotic patients. These ROIs’ gray matter deficits cause such functional abnormalities (113).

Nakamura et al., found structural deviations in multiple brain areas with the help of structural brain measures in SZ patients (16). Kawasaki et al., assessed the pattern of the eigen image from sMRI and found lower gray matter concentrations in the bilateral medial frontal regions, bilateral lateral frontal regions, bilateral insular regions, and left temporal region (17). Ulas et al. found four ROIs from the two hemispheres of the brain summing up to a total of eight different brain regions: Amygdala, Entorhinal Cortex, Superior Temporal Gyrus, and Thalamus which were found to be impaired in SZ patients (19) and discovered six different brain regions: Dorsolateral prefrontal cortex, Entorhinal Cortex, and Thalamus which are found to be impaired in SZ patients (20).

An abnormality of hippocampal asymmetry was also identified in the SZ subjects (23). GM volume reduction was the proof in the frontotemporal cortex (24). The frontal regional abnormality noted is reliable with the clinical appearance of the SZ (27). Morphological patterns analyzed and WM, GM, CSF volumes are identified (32). Some discriminative patterns such as GM appeared in the precentral, postcentral, superior frontal and temporal, parahippocampal gyri and cingulate (33, 35, 112).

Takayanagi et al., discovered that there was a great GM volume reduction of the superior temporal gyrus, which is one of the most consistently reported abnormalities in the brain structure of SZ patients (37). Koutsouleris et al., investigated prefrontal perisylvian and subcortical brain structures and found the structural alterations located primarily in the right ventrolateral prefrontal, insular, and superior temporal cortices (116). Castellani et al., discovered dorsolateral prefrontal cortex (DLPFC), a reliable neuroanatomical marker of the disease and found out some variations in this region (140). Based on the reduced GM densities (from Voxel-based Morphometry) in the frontal and superior temporal lobes and hippocampus and decreased GM densities in in basal ganglia and left occipital lobe in SZ patients with respect to normal controls (141).

Ota et al., discriminated the female SZ with female normal subjects with GM and CSF volume obtained with voxel‐based morphometry. GM volume reduction was found in bilateral insulae–superior temporal regions and medial frontal gyrus and CSF dilatations were found in the third ventricle, bilateral Sylvian fissures, and around the prefrontal regions in the patient group (119). In another study, GM variations were found in the dorsolateral prefrontal cortex (DLPFC) or occipital cortex (OC) (38, 140, 142).

Zanetti et al., indicated reduced GM morphometric abnormalities in the bilaterally the dorsolateral and orbital frontal cortices, temporal cortex and insula, and also the left posterior cingulate cortex. Increased GM volume was observed in the right anterior cingulate cortex of SZ patients and Enlargements of the 3rd ventricle and the posterior (occipital) horn of the left lateral ventricle were also contributing to the diagnosis of first-episode SZ (143). Moura et al., discovered differences on the volume of cerebrospinal-fluid, right cerebellum cortex, bilateral thalamus, bilateral parahippocampal gyrus, middle anterior and central part of corpus callosum, left superior frontal gyrus, left hippocampus, right supramarginal gyrus, left plexus choroid and right medial orbitofrontal cortex (144).

Chin et al., investigated the variations of GM densities from the following regions and may be useful for clinical approaches of SZ: the occipital fusiform gyrus, middle frontal gyrus, pars opercularis of the inferior frontal gyrus, anterior superior temporal gyrus, superior frontal gyrus, left thalamus and left lateral ventricle (42).

#### fMRI

Biological markers of fMRI can be seen in seven brain networks, such as, Default Mode, Parietal, Lateral and Medial Visual Cortical, Dorsal Visual Stream, Medial frontal, Temporal (29). Temporal lobe and default mode networks were reliably identified in all SZ patients (30, 52, 55–57, 64, 69, 70, 74, 145–149).

The functional network connectivity between the brain regions of the frontal cortex and cerebellum were indicated the highest discriminative power in rs-fMRI, which is used to test the hypotheses that synchronicity across anatomically-defined brain regions or functionally-hypothesized networks are different, because of age, disease, or the task being performed (60, 64, 68–70, 73, 75, 99, 144–148, 150). Most of the researchers used resting state fMRI to find the brain alterations and functional connectivity (42, 43, 53, 54, 56, 58, 59, 67, 68, 71, 72, 80, 81, 83, 98, 144–146, 148, 151–159) and others used some task-related (28, 55, 57). Cognitive control tasks were performed in some studies and assumed a means of best solution to classify SZ with fMRI data (62, 160, 161). Functional connectivity measures such as regional homogeneity (ReHo), amplitude of low frequency fluctuations (ALFF), and fractional amplitude of low frequency fluctuations (fALFF) used to discriminate SZ and normal subjects (72, 76, 153, 162). Some of the researchers used Auditory Odd Ball task to capture the functional connectivity in the brain regions (28, 57, 63, 67).

Many works have reported that SZ demonstrate dysfunction of the prefrontal cortex during the performance of working memory tasks (62, 147, 163). Costafreda et al., investigated with the neural responses to verbal fluency and showed the increased activation in the anterior cingulate, left dorsolateral prefrontal cortex and right putamen as compared to healthy controls, as well as reduced deactivation of precuneus and posterior cingulate (55). Du et al., showed the features extracted from Default Mode Network (DMN) and motor-temporal components lead to significantly high classification accuracies in discriminating controls and patients in the AOD task (57).Venkataraman et al., discovered the co-existing patterns of increased connectivity between frontal and parietal regions, and decreased connectivity between temporal and parietal regions, and between the temporal cortices bilaterally. This work revealed the information of abnormal patterns of functional connectivity that the positive symptoms related to decreased parieto-temporal connectivity and negative symptoms related to increased fronto-parietal connectivity (59). SZ has been associated with aberrant functional connectivity (FC) within and between the DMN, the Central Executive Network (CEN) and the salience network (SN) (162).

Yu et al., classified the functional dysconnectivity with high discriminative power with three types of signatures, namely state connectivity patterns, trait connectivity patterns, and compensatory connectivity patterns. From this study, functional connectivity between the cerebellum and the prefrontal lobe, the middle temporal gyrus, the thalamus and the middle temporal poles exhibited high discriminative power for the trait patterns. The connectivity related to the right precuneus, the left middle temporal gyrus, the left angular and the left rectus showed higher discriminative power for the compensatory patterns. In the state patterns, several brain regions exhibited greater weights in the following regions., the hippocampus, the ACC, the PCC, the medial prefrontal cortex, the middle temporal gyrus, the parietal gyrus and some cerebellar regions (61).

Anticevic et al., showed the Thalamo-Cortical Disturbances in SZ. It was associated with significantly reduced prefrontal–cerebellar–thalamic coupling, but also increased coupling with all bilateral sensory–motor cortices. This work found robust reductions in thalamo-prefrontal–striatal–cerebellar coupling, but also a symptom-related increase in thalamo-sensory–motor coupling in SZ (161). Watanabe et al., found that functional connectomes reside in a high dimensional space, and recovered altered connectivity between frontoparietal network and default mode network, which is an important brain network involved in autobiographical memory and internally generated mental simulations (65). Cheng et al., discovered an increased thalamus–primary somatosensory cortex connectivity was the most noteworthy abnormality in SZ patients; a number of thalamic links with motor and sensory cortical regions showed increased connectivity in SZ, whereas thalamo–frontal connectivity was weakened (66).

Chyzhyk et al., used rs-fMRI and testing two kinds of features - functional connectivity measures computed by lattice auto-associative memories (LAAM), and local activity measures, such as ReHo and fALFF. Functional connectivity measures seeded in right Heschl’s gyrus consistently showed stronger discriminative power and ALFF showed poor discriminative ability. fALFF achieved 100% accuracy, sensitivity, and specificity and performed significantly better than ReHo (p<0.01), which achieved 97% accuracy (67). SZ has been found with abnormal functional connectivity (FC) within and between the default mode network, the central executive network and the salience network (162). There was preliminary evidence for more consistent intrinsic functional brain connectivity changes with cortical networks for medial temporal lobes and cerebellum than for striatum and amygdala in SZ (68).

The lower Voxel-mirrored homotopic connectivity (VMHC) in the precuneus, fusiform gyrus/cerebellum lobule VI, and lingual gyrus/cerebellum lobule VI and SZ patients have decreased homotopic connectivity in the motor and low-level sensory processing regions (69). The ReHo, fALFF and seed-based Granger causality analysis (GCA) maps were given into a multiple kernel learning classifier, to determine whether patterns of local and effective connectivity can differentiate controls from patients. The inflow and outflow of Granger causal information between visual cortex and thalamus is affected in SZ (72). Chatterjee et al., reported several brain regions like cerebellum, Heschl gyrus, vermian, inferior temporal gyrus, superior temporal gyrus, superior frontal gyrus, insula, and amygdala that have been reported in the dysfunction of the working memory with SZ patients (75).

Modinos et al., investigated impairments in measures of emotional functioning parallel to SZ and revealed a distributed and subtle set of alterations in brain function within the emotional circuitry of individuals with high PP, providing neurobiological support for the notion of dysfunctional emotional circuitry in this group particularly in medial prefrontal cortex, anterior cingulate cortex, insula and amygdala (149). Zhao et al., found that atriatum, cerebellum, and dorsolateral prefrontal cortex were recognized as the most group-discriminative brain regions and dorsal striatum (DLPFC), has been proved to play a vital role in the pathophysiology of SZ (84).

Zhang et al. reviews key structural and functional MRI techniques applied to antipsychotic-naïve first-episode SZ (FES) and neuroimaging biomarkers in China. This work identifies candidate imaging biomarkers such as GM reduction within temporo-frontal regions and cerebellum, altered brain function at cerebello-cortical connections and default mode network. These biomarkers target disease-inherent effects (medication-free), supporting clinical translation through multicenter validation (164).

#### Perfusion MRI and diffusion MRI

Most frequent biomarkers for DTI are Functional Anisotropy (FA), Relative Anisotropy (RA), Mean Diffusivity (MD), WM and GM perfusion indexes and mean of blood flow values, such as, relative Cerebral Blood Volume (rCBV), regional Cerebral Blood Flow (rCBF) and Mean Transit Time (MTT). In DTI, most of the studies found reduced functional anisotropy as one of the biomarkers (87–94, 97, 160, 165).

RA is a DTI metric that particularly measures the degree of anisotropy. It is calculated by comparing diffusion’s anisotropic (directional) and isotropic (non-directional) components. It reflects how much water molecules in a certain location of the brain preferentially diffuse in one direction rather than evenly in all directions. Mean diffusivity is a numerical indicator of the average diffusion of water molecules within a voxel (3D pixel) of brain tissue that is used to determine WM integrity. While MD shows the total diffusion, FA reflects the direction of diffusion. FA and MD are frequently tested jointly to obtain a more complete picture of white matter integrity.

rCBF is the rate of blood flow to specific parts of the brain. It’s a test used to evaluate brain activity and function, usually in relation to cognitive processes or neurological illnesses. Neuroimaging commonly employs rCBV and dynamic susceptibility contrast (DSC) magnetic resonance imaging. MTT is a critical metric in perfusion imaging that measures blood flow and is used to identify areas of reduced blood flow (hypoperfusion) in diseases such as stroke. It is derived by dividing CBV by CBF. MTT is commonly measured with perfusion MRI techniques like as dynamic susceptibility contrast (DSC) MRI. The most distinctive brain regions from the fused measures of WM such as FA and MD showed the discrimination between SZ and control subjects in the SZ. In relation to WM assessment, fractional anisotropy of the left corticospinal tract had highest feature importance in multimodal feature integration (160).

Kubicki et al., planned to assess diffusion anisotropy between SZ patients and control subjects and calculated RA. This RA maps reveal visual contrast between GM and WM regions of the brain. Increased diffusivity and reduced FA within temporal and prefrontal lobes, and abnormal within the fiber bundles connecting these regions are the findings in SZ (87). Kannan et al., found reduced FA in the regions of Frontotempo and splenium, anterior cingulum, bilateral CC, AC, MTG, PHG, left STG and reduced bilateral right and frontal occipital FA (88).

Pinkham et al., used the quantitative assessment of CBF with magnetically labeled arterial blood water as an endogenous tracer. Increased blood flow found in the regions of right middle temporal gyrus, left putamen/superior corona radiata, cingulate gyrus, and superior frontal gyrus, associated with SZ. Reduced CBF was found in the middle frontal gyrus and precentral gyrus (89). Caan et al., found higher FA in the most of the WM, especially posterior limb of the internal capsule and uncinate fasciculus and reduced FA in the corpus callosum regions (91).

Ingalhalikar et al., and Ardekani et. al., used DTI with the extracted features as mean FA and MD measures for all regions to discriminate normal subjects with SZ. These abnormality measurements represent the diagnostic and prognostic biomarker in SZ (93, 95). Squarcina et al., used pMRI to assess the mean values of blood flow and its volume in the regions of parietal, occipital and temporal lobes, insula, left and right frontal, cerebellum and caudate, to discriminate SZ and normal subjects. rCBV, rCBF and MTT were calculated to assess the reduced blood flow and volume and these represent WM and GM perfusion indexes. Reduced blood flow and volume detected were found in the left and right frontal lobes, left cerebellum and right caudate regions (96).

#### PET scan

PET scans are significant in neuroimaging because they provide unique insights into brain activity and metabolism, allowing diagnosis of illnesses before structural changes become obvious with conventional imaging modalities. This is also used to detect functional connectivity in the brain regions (99). Levy et al., focused on the cortical/subcortical spatial pattern in two directions, anterior/posterior and chiasmatic and found the brain dysfunction of regional glucose metabolism with SZ patients with and without photographic image tracking task (98).

Josin et al., measured regional cerebral blood flow using PET with the oxygen isotope O and used functional connectivity images for all patients in voxel level. This work measured regression coefficient, which represents the degree to which predicted variations of cerebral blood flow in the cardinal frontal regions. This coefficient is used as a functional connectivity measure, covariance between cerebral activity in that voxel and cerebral activity in the frontal ROI (99). Bose et al., proved the Striatal dopaminergic overactivity is one of the factors in diagnosing SZ and used 18F-Fluorodopa or FDOPA, which is a radioactive agent used in PET diagnostic test. This work focused on FDOPA rate constants within the anterior-posterior subdivisions of the striatum, and compared with the model with a general linear analysis (100).

#### Challenges of biomarkers

Biomarkers are objective biological indicators that can predict clinical outcomes, resulting in precision medicine. The key problem of biomarker creation is that the typical disease categorization in the correct classification is based solely on specific symptoms, which causes patients to become confused with different biomarkers.

Clinical challenges with SZ include subjective diagnosis, experiential treatment, and poor prognosis. In recent years, various intriguing discoveries have been made in the investigation on objective biomarkers of SZ, with a particular emphasis on genetic susceptibility genes, metabolic indicators, immunological indices, brain imaging, and electrophysiological characteristics.

For reliable clinical utility, researchers must first determine the accuracy of biomarkers, and then consider using biomarker information by considering relevant risk benefits and clinically meaningful outcome improvement when managing patients.

## Discussion

## Classification and detection of SZ using ML models

The classification of SZ using ML models has emerged as a promising approach to improve early diagnosis, treatment personalization, and understanding of the disorder. SZ is a complex psychiatric condition characterized by heterogeneous symptoms, making traditional diagnostic methods challenging and often subjective. ML techniques leverage large-scale data, including neuroimaging (fMRI, sMRI, DTI), genetic profiles, clinical assessments, and cognitive performance measures, to identify subtle patterns and biomarkers associated with the disorder.

Commonly used models include SVM, RF, Logistic Regression (LR), KNN. These models help in binary classification (SZ vs. HC) and multi-class classification (different stages or symptom subtypes). Feature extraction and dimensionality reduction methods, such as PCA and ICA, are often applied to handle high-dimensional neuroimaging and genetic data and which have achieved good classification accuracies. Finding structural abnormalities with sMRI, SVM is the best ML model which is proven by many researches (19, 32–34, 36, 37, 40, 69, 79, 101, 115, 116, 139, 166, 167). Most of the researchers have used and trusted SVM is one of the best classifiers in SZ detection with fMRI data (42, 67, 68, 71, 73, 75, 98, 153, 155).

Chin et al. utilized SVM with spatial and anatomical regularization to classify SZ in sMRI scans, identifying seven key ROIs: the left thalamus, left lateral ventricle, pars opercularis of the inferior frontal gyrus, anterior superior temporal gyrus, middle frontal gyrus, and superior frontal gyrus (69). Algumaei et al. explored four types of resting-state fMRI biomarkers and applied both feature-level and decision-level fusion techniques to distinguish between SZ patients and HC. Their feature-fusion method achieved an impressive accuracy of 98.71%, while decision-level fusion reached 97.85% (77). The use of fMRI data for SZ classification has gained traction due to its ability to reveal functional connectivity. Researchers often construct “networks” to investigate brain function. Using group ICA and Pearson correlation coefficients, one study achieved 92.9% accuracy in classifying SZ versus HC, demonstrating the effectiveness of these advanced analytical methods (78).

The DPCA method is beneficial for dimensionality reduction prior to classification into two groups and has a computational speed comparable to that of conventional PCA. The reason for this is that the primary computational task in PCA involves determining the eigenvectors of a covariance matrix, and DPCA utilizes the same eigenvectors. When DPCA was used on diffusion tensor-based fractional anisotropy images, the classification error with 60 components approached the minimum error, and the Mahalanobis distance was twice as large with DPCA compared to PCA (92). Ingalhalikar et al. used non-linear SVM used to discriminate SZ with human control using anisotropy features and getting good accuracy (93). Ardekani et al. used DTI images with two biomarkers MD and FA abnormality scores for classification and achieved a good accuracy as 94% for FA Measures and 98% for MD measures with the help of ML algorithm Fisher’s LDA (95). From DTI data analysis, most of the researchers used ML algorithms to discriminate SZ and HC, preferably SVM.

Yang et al., applied the ICA and SVME classifier for SNP + fMRI data, achieved 87% accuracy when using multimodal data classification (53). Du et al. achieved 98% accuracy in the auditory oddball task and 93% in rest data by extracting features from resting state and task-based fMRI, using a combination of a two-level feature identification scheme with kernel principal component analysis (KPCA) and Fisher’s linear discriminant analysis (FLD) (57). Cetin et al. suggest a novel framework for classifying patients with SZ that utilizes both fMRI and MEG to examine functional network components in the resting state through the ICA method. This study found a 5.12% increase in classification accuracy when using combined data (128). Qureshi et al., had taken the features of Structural ROI measures, Global functional connectivity from sMRI and fMRI data and produced the accuracy of 99.29% with ELM (129).

The results indicate that SVM and RF are frequently used on structured clinical and neuroanatomical records, as well as MRI data, with SVM and logistic regression often yielding the best performance for neuroanatomical features. It is becoming more common to recommend incorporating into clinical workflows for SZ care ML-driven diagnostics, which can enable early detection and personalized treatment planning.

Research findings indicate that ML models can attain high accuracy, sensitivity, and specificity, frequently surpassing traditional diagnostic methods. Challenges persist, including dataset imbalance, small sample sizes, variability in clinical presentations, and the absence of standardized features across studies. It is important to also consider ethical aspects related to patient privacy, the interpretability of ML models, and possible biases.

To sum up, ML-based classification of SZ offers a useful supplement to clinical assessment, with the potential to facilitate objective, data-driven, and individualized psychiatric care. Future research necessitates the use of larger multimodal datasets, explainable AI methods, and incorporation into clinical workflows to guarantee reliability and acceptance in practical healthcare environments.

## Classification and detection of SZ using DL models

The use of DL models for the classification of SZ has gained significant attention due to their ability to automatically learn complex, non-linear patterns from high-dimensional biomedical data. Unlike traditional machine learning, which relies heavily on handcrafted features, DL models can process raw or minimally preprocessed data from sources such as sMRI, fMRI, EEG signals, genetic data, and clinical records.

Commonly applied architectures include CNN for spatial neuroimaging data, RNN and LSTM networks for sequential EEG and clinical time-series data, and Autoencoders or Variational Autoencoders (VAEs) for unsupervised feature learning and dimensionality reduction. More recently, Graph Neural Networks (GNNs) and Transformer-based models have been employed to capture brain connectivity patterns and multimodal integration.

It can be seen that over the last decade, research in the area of SZ detection has slowly been turning towards the developments with DL models. However, ML approaches also played and still pay a significant role in SZ prediction. Most of the researched had chosen DL model for SZ classification with the multimodal data (98, 109, 125, 133–135, 137, 138, 153, 168). Very few researchers used ML models for the prediction of SZ with SMRI data (44, 49–51, 82, 85, 169).

Han et al. utilized feed-forward networks and three-stage DL networks to classify SZ from resting-state fMRI data, achieving a prediction accuracy of 79.3% (80). Similarly, Zeng et al. developed a deep discriminant autoencoder model using multisite fMRI data, identifying an integrated cortical-striatal-cerebellar circuit. Their findings showed an accuracy of 81% for leave-site-out transfer classification and 85% for multi-site pooling classification, suggesting that disruption in this circuit may link cognitive deficits and psychosis in SZ (81).

Oh et al. distinguished SZ Spectrum Disorders from healthy controls using a CNN trained on task-based fMRI data, achieving an accuracy of 84.43% (82). However, this accuracy still falls short for clinical applications, although significant areas for classification, like the inferior and middle temporal lobes, were identified. Yan et al. explored fMRI data using multi-scale RNN for the first time. They reported accuracies of 83.2% and 80.2% for multi-site pooling and leave-site-out transfer classifications, respectively. Limitations included insufficient data on medication effects and residual influences from head motion despite preprocessing (83).

Hu et al. utilized two distinct 3D sMRI datasets and employed both linear and non-linear SVMs, alongside a 3D CNN model. Their findings revealed that the 3D CNN model significantly outperformed traditional methods by a 10% accuracy margin, enhancing the potential for individual-level diagnosis in psychiatric conditions (45).

Zhao et al. proposed a hybrid DL model that integrates temporal coherence and dynamics of brain activity using Convolutional RNNs, DNNs, and SVMs. This innovative approach identified crucial brain regions for predicting SZ, including the striatum, dorsolateral prefrontal cortex, and cerebellum (84).

Masoudi et al., obtained 99.35% of accuracy with 3D CNN model applied to sMRI+fMRI+DTI fusion features (133) and got 99.5% of accuracy with Gated Recurrent Unit with 2D CNN, 3D CNN applied to fMRI+DTI data (134). Srinivasagopalan et al., performed SZ classification with DNN applied to sMRI and fMRI and got an accuracy of 94.4% (138). Algumaei et al. explored four types of resting-state fMRI biomarkers and applied both feature-level and decision-level fusion techniques to distinguish between SZ patients and HC. Their feature-fusion method achieved an impressive accuracy of 98.71%, while decision-level fusion reached 97.85% (77).

Rahaman et al., proposed combining sMRI, fMRI and SNP data in a multimodal classification framework and produced 92% accuracy in SZ prediction and outperformed with the other DL models applied to unimodal and multimodal data (109). Similarly, Kanyal et al., applied to the same multimodal data as Rahman et.al., and obtained the accuracy of 79% by using DenseNet model (139).

Studies report that DL models often achieve superior classification accuracy compared to conventional ML approaches, effectively distinguishing patients with SZ from healthy controls and, in some cases, differentiating between subtypes or stages of the disorder. Importantly, multimodal DL frameworks that combine neuroimaging with clinical or genetic data show enhanced performance and robustness.

Reported diagnostic accuracies range from 78-99% with the highest scores on large, multicenter studies using ensemble or multimodal DL models. DL models outperform conventional ML models and allow for more precise and sensitive detection, localization of predictive regions (subcortical structures and ventricles), and adaptability to new datasets (50).

Even with these advancements, challenges remain, such as the small size of datasets, significant danger of overfitting, low interpretability of DL models, and the necessity for explainable AI to establish clinician trust. Moreover, differences in imaging protocols and demographic factors can influence the generalization of the model. In summary, classification based on DL provides an effective means for objective, adaptive, and scalable solutions and data-driven diagnoses of SZ, paving the way for early detection and individualized treatment.

## Multimodal integration

Multimodal integration is applied in schizophrenia prediction since schizophrenia does not localize itself in one brain anomaly but includes several abnormalities, namely the presence of structure, function, and connectivity alterations. The use of only one imaging method is insufficient to take into account all these alterations, and therefore predictive accuracy will not be high.

The fundamental notion behind such an approach is dysconnectivity theory of schizophrenia. It postulates that schizophrenia emerges due to dysfunctional connections between brain structures rather than any local brain problems. Various imaging methods are involved in revealing various abnormalities related to schizophrenia. Specifically, the use of sMRI can detect atrophy of gray matter, fMRI can show abnormalities in networks and connectivity patterns, whereas DTI highlights abnormalities in white matter tracts. Multimodal fusion helps in understanding cross-modal relationships that further enhance the ability to classify and predict.

In terms of ML and DL, multimodality enhances robustness and generalization. Datasets for schizophrenia are typically noisy and heterogeneous, and multimodal fusion makes the models less dependent on noisy information and more robust in finding biomarkers. This becomes even more critical when we think about transferring such models to clinical settings, where subject-to-subject variance is significant.

Finally, multimodal modeling matches well with the objectives of precision psychiatry, which seeks to go from diagnostic criteria based on symptoms to biological predictors. For instance, by fusing neuroimaging information with clinical or cognitive features, we can develop personalized models to predict the progression of a certain type of disease (170).

Recent studies in schizophrenia prediction have concentrated on multimodal neuroimaging integration, amalgamating alternative methodologies including structural MRI (sMRI), functional MRI (fMRI), diffusion tensor imaging (DTI), and electroencephalography (EEG). Each modality captures unique neurobiological attributes: sMRI assesses cortical thickness and grey matter volume, fMRI delineates functional connections and network dynamics, and DTI examines white matter integrity via metrics like fractional anisotropy. Combining these many types of data gives a better picture of brain problems that are linked to schizophrenia. These problems are known to affect structural, functional, and connection areas (the dysconnectivity hypothesis for schizophrenia) (170, 171).

However, there are still several issues that should be addressed. First of all, multimodal databases tend to have a small number of samples because the process of collecting different imaging data for the same patients is very complicated and expensive. Also, variations in data acquisition methods, pre-processing procedures, and features’ dimensionality create additional sources of heterogeneity. Moreover, multimodal analysis imposes considerable computational requirements, especially when deep learning techniques are applied.

Nonetheless, multimodal imaging is regarded as one of the promising areas in which to invest future schizophrenia studies. In the future, researchers must develop effective fusion approaches such as those at the feature level, the model level, and hybrid ones. In addition, they must pay attention to enhancing cross-site harmonization methods and improving explainability for the successful transition into the clinical practice domain. Domain invariant learning, harmonization, and architecture scalability are critical approaches to enhance generalizability for predicting schizophrenia using multimodal methods across different cohorts (172).

## Challenges and limitations

**Figure 3:**
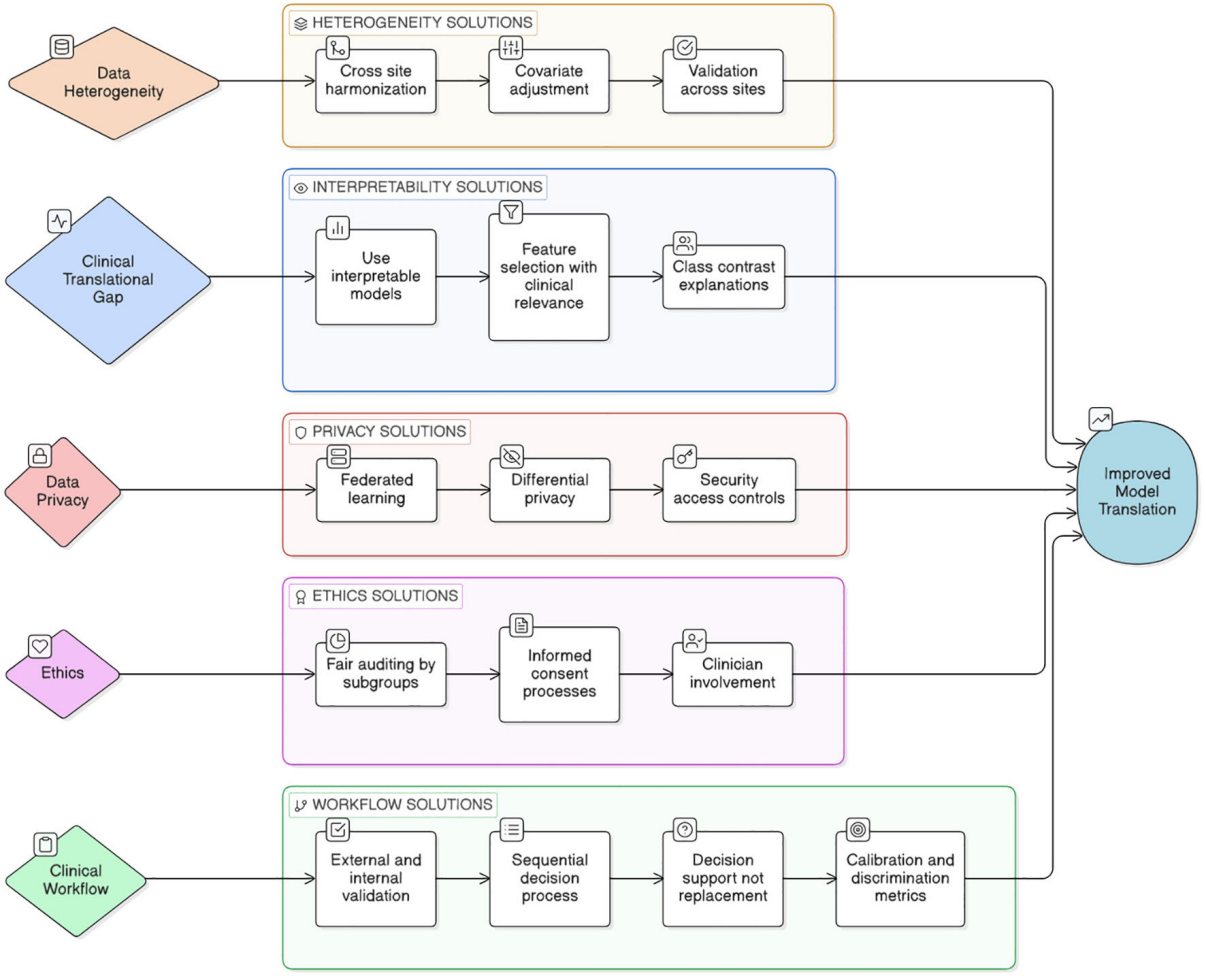
Challenges in SZ prediction and its solutions.

Medical imaging methods, including sMRI, fMRI scans and other scans, might differ according to the quality of the equipment employed, the scenarios under which they were obtained, and the prior experience of the person interpreting the findings. Similarly, electronic health records might be unreliable or poorly organized, making it difficult for DL to generate accurate predictions. One of the key challenges in applying ML/DL to SZ detection is the lack of large, well-labeled, diverse, and high-quality datasets. To allow for prompt intervention, models must be capable of early detection, even before the entire start of the symptoms. Early detection is difficult due to the intricacy of early symptoms and the scarcity of precise biomarkers. Most of the present ML/DL designs, particularly DNN, are classified as “black-box” techniques. A lack of transparency is an essential issue in healthcare applications, where recognizing the reasoning behind predictions is vital for clinical success. Addressing legal, ethical, and regulatory issues is necessary when implementing ML/DL models in practical contexts. The main challenges to SZ prediction are depicted in the **Figure 3**.

According to Zhang et al., biological subtyping of heterogeneous psychiatric conditions allows for specific drug development and tailored interventions. In syndromes that are common, such as schizophrenia and mood disorders, there exists biological variability that can only be understood through biological subtypes rather than the severity dimension. The discovery of these subtypes allows for targeted and precise modifications of the mechanisms involved, thus going beyond mere symptomatic interventions. By utilizing methods such as neuroimaging and peripheral biomarkers, researchers have been able to define specific groups that differ in the presence of biological traits that can form the basis of new drug development initiatives. There remains the need to enhance the reliability and validity of biological subtypes and understand their clinical, developmental, and psychosocial characteristics (173).

### Data heterogeneity

Schizophrenia has large heterogeneity in symptoms, disease state, and manifestations; therefore, a high accuracy observed in a particular dataset will not generalize to other datasets. Classical case-control models can be promising in a particular dataset but can fail in other datasets because they can learn features unique to that specific dataset. Despite the promising findings that have been presented in terms of high classification accuracies when using machine learning algorithms to classify patients with SZ, one needs to remain cautious about their significance due to the heterogeneous nature of the condition itself. Various factors such as the differences in symptoms, the phase of the disease, drug use, demographics, signal acquisition method, pre-processing, and site-specific methods may influence how accurate the models can perform in reality. Degradation in performance arises in cases where models developed using institution-specific data show poor results with respect to other data. For example, brain image classifiers perform at 73 percent accuracy within institutions but show accuracy at 55 percent with other institutions’ data (174). Overfitting is the main cause of such scenarios because models learn to pick up noises rather than the biological signals in the dataset.

The best results are often obtained by conducting tests on independent datasets, minimizing site or batch differences, and avoiding uniform models (175). To address the problem of generalizability in schizophrenia prediction, it is important to emphasize validation, cross-site harmonization, covariate adjustment, and model training with heterogeneity in mind.

The above approaches prevent overfitting to the artifacts of the particular cohort and increase the chances of replicating the results in an independent clinical setting. The following methods should be used when transferring the model to another clinical environment because each clinical environment differs in terms of patients’ demographics and data quality (176).

### Clinical translational gap

Although many schizophrenia prediction models have demonstrated excellent classification results, there are still major obstacles that stand in the way of implementation in clinical practice. The main challenges here are insufficient external validation of models, biases associated with choice of patient cohorts, problems with interpretation of complex models, risks connected to privacy concerns over mental health and neuroimaging data, as well as ethical aspects of such applications. Furthermore, training data are frequently gathered in research conditions rather than reflecting actual clinical realities. This resonates well with the research that indicates that only a few psychiatric prediction models have been validated and that their implementation remains restricted due to issues such as clinical trust, availability, and interpretability of data (176). There are some bench-to-bedside barriers in clinical translation of SZ prediction.

#### Bench-to-bedside barriers

##### Interpretability

Most schizophrenia models have been created using deep learning techniques or ensemble models that can be considered black box models where understanding how predictions are generated is not easy. For a psychiatrist, this will be more of a problem compared to practitioners in other disciplines due to the nature of mental disorders that are characterized by heterogeneity and complexity.

To address this issue, one can use:

- Interpretable models when accuracy is equivalent, like logistic regression, decision trees, and sparse models, since they are more straightforward for clinicians to understand (177).
- Feature selection and clinically motivated features that allow the model to depend on relevant biomarkers instead of random associations (178).
- Class contrast or instance-based explanations to demonstrate the differences between the patient and other cases (177).

##### Data privacy

Data privacy is a real barrier in SZ prediction because the models often rely on highly sensitive data such as speech, behavior, clinical notes, and neuroimaging, and patients may be reluctant to share it if confidentiality is unclear. Psychiatric and neuroimaging data can reveal identity, symptoms, or relapse risk, so leakage can have serious consequences. Trained models can sometimes memorize private information, so sharing model weights can also be risky (179).

The best way to address it is to combine privacy-preserving methods with clear governance, minimal data collection, and patient-centered consent. Federated Learning (FL) is widely promoted as a privacy-preserving training method, enabling decentralized model training without directly sharing patient data. Although conceptually attractive for mental health settings, FL alone offers weak privacy guarantees and remains susceptible to gradient leakage attacks (179).

Other popular solutions include (179):

- Implementing differential privacy or secure aggregation methods to decrease risk of gradient/model exposure.
- Creating security mechanisms where only the proper personnel have access rights.
- Using federated learning where data remain local on the hospital/device level and only updates are exchanged.

##### Ethics

Ethical issues still play a crucial role when translating predictive models for schizophrenia into practice. They are related to algorithmic bias, disparity in performance, insufficient informed consent, potential stigma, and transparency. To prevent such ethical pitfalls, research should present performance metrics by subgroups, perform fair auditing, provide clinician involvement, acquire informed consent, and govern data use and model implementation effectively (179).

### Clinical workflow

The clinical implementation of predictive models for schizophrenia suffers not only from the issue of accuracy but also from issues of practicality and the quality of validation. Most models rely on data that cannot be feasibly collected in a regular clinical setting, and internal validation is inadequate to guarantee success in a clinical setting. In the future, emphasis must be placed on external validation, calibration, decision-curve analysis, and staged workflows combining multiple forms of data. Major workflow issues are data collection burden, poor validation, dependency on threshold-based output.

To address this issue, one can (180, 181)

- Ensure external validation and validation both externally and internally to demonstrate that the model is generalizable beyond one specific group.
- Ensure that the model is incorporated within a sequential decision process where less burdensome predictors precede other tests when necessary.
- Develop the test as a decision support system rather than a replacement of the psychiatrist’s expertise in decision-making.
- Provide estimates of calibration as well as discrimination because a good predictive model must be well calibrated.

### Clinical context with AI approaches

Apart from diagnosis, image-based applications of artificial intelligence in schizophrenia have been studied in order to predict future treatment responses. Whereas some algorithms are intended to enhance diagnostic ability or improve early diagnosis, other AI models seek to facilitate precision psychiatry by finding patients who would not be able to benefit from the treatment or by pinpointing neural markers that could assist in personalized treatment. Therefore, when evaluating the clinical utility of imaging-AI models, it is important to understand their purpose.

The linkage between brain systems and the corresponding human behavior is a very important step in pinpointing the targets of therapeutic interest. But understanding brain-behavior relationships for application purposes remains a persistent problem, involving many different issues (182, 183).

The application of knowledge about brain and behavior correlations into clinical settings is plagued by obstacles at various stages, including identification, interpretation, and implementation. This process of converting neuroimaging data to practical psychiatric treatment solutions is thus hindered. The literature review categorizes obstacles according to different stages, namely, the identification stage (through multimodal imaging and psychometric techniques), the interpretation stage (concerns over causal relationships and weak correlations), and the implementation stage (problems of technology, politics, and ethics).

#### Identification problems

Several technical problems exist in the identification of brain attributes with behaviors through the use of multimodal imaging and psychometrics.

#### Interpretation problems

The major challenges faced include the establishment of a model of brain behavior interactions, causality in the interactions, and weak statistical association.

#### Implementation obstacles

Some of the obstacles that stand in the way include technical problems, political obstacles, and ethical considerations.

Clinical prediction models for schizophrenia are currently faced with issues in attaining accurate results, as well as external validity and implementation for vulnerable individuals. Almost all aspects of the prediction model have limitations in practice, which prevent it from being used independently as a tool for diagnosing schizophrenia (111, 182, 184, 185).

Brain-behavior associations are clinically relevant in schizophrenia because they may support both diagnostic optimization and treatment planning. In some settings, imaging-AI models are used to improve early detection or diagnostic classification, whereas in others they help estimate symptom severity, functional outcome, or treatment response. Therefore, the clinical context of use should be explicitly stated when discussing prediction models, since the same biomarker may have different value depending on whether the goal is diagnosis, prognosis, or therapy selection.

### ML challenges and limitations

**Figure 4:**
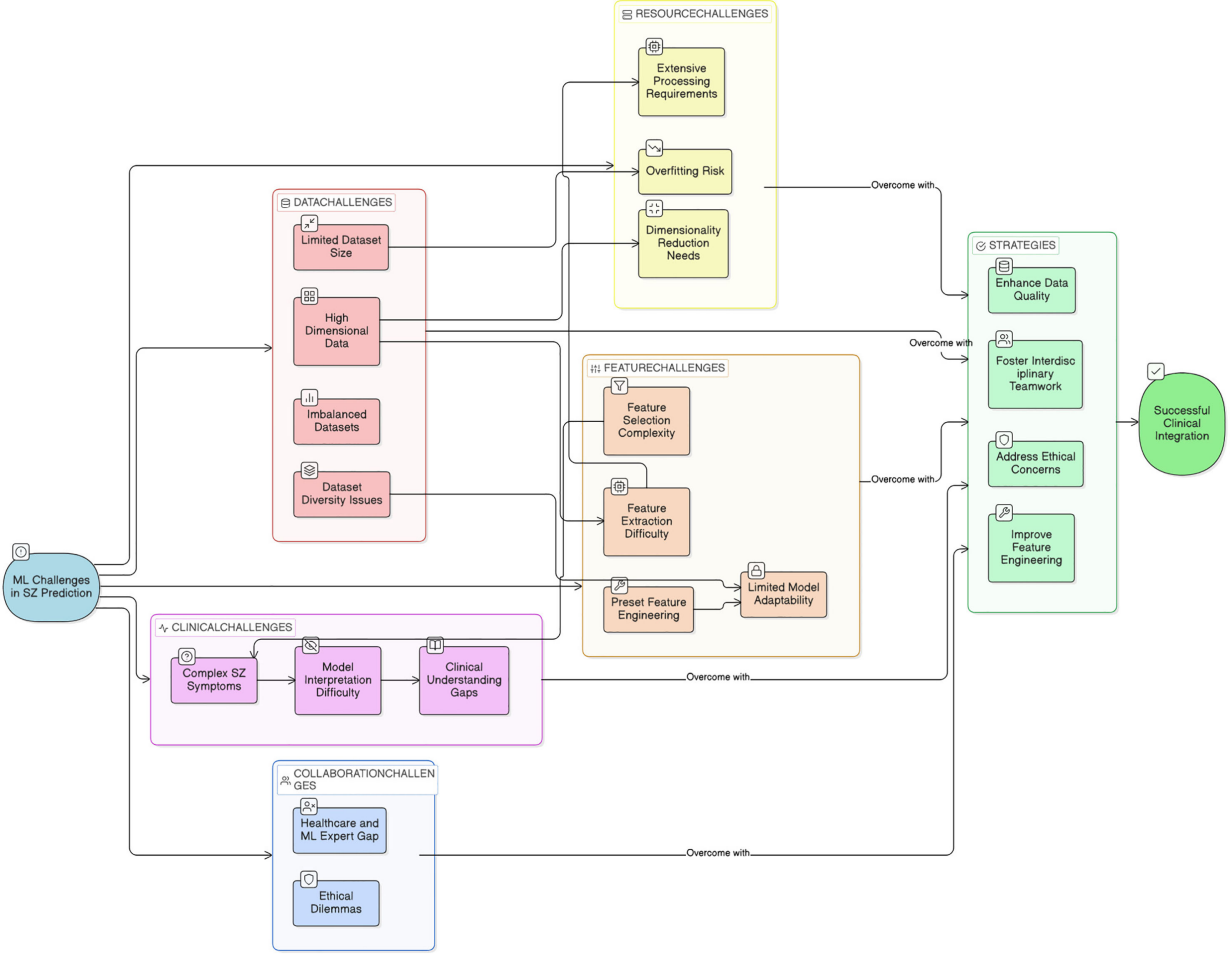
ML challenges in SZ prediction and its solutions.

Despite significant advancements in SZ prediction using medical imaging, several challenges and limitations persist. The diversity and limited size of datasets hinder the development of robust ML models for SZ classification. Additionally, the complexity of SZ symptoms complicates the selection of relevant variables and the interpretation of model outputs, affecting clinical understanding. Other challenges include imbalanced datasets, ethical dilemmas, and the need for collaboration between healthcare professionals and ML experts. Overcoming these barriers is crucial for successfully integrating ML into clinical diagnostics. Strategies to enhance data quality, foster teamwork, and address ethical concerns are essential. **Figure 4** illustrates the common ML challenges and limitations with possible solutions.

Traditional ML methods follow a multi-stage process of feature selection, extraction, and preprocessing. According to Cortes-Briones et al., task-specific features are commonly used to build ML models, which often rely on preset feature engineering (104). This approach can limit the model’s adaptability to different data types and applications, making it less autonomous in the learning process. While ML provides a structured framework, it may struggle with complex data relationships due to its reliance on explicit feature engineering.

Although the ML models are effective in detecting SZ, they all have certain limitations. First, short or limited datasets may lead to overfitting, a phenomenon where the model performs well on training data but poorly on unknown data. Second, extracting the most relevant characteristics from high-dimensional data, like neuroimaging, can be challenging but is essential for model performance. Third, extensive processing resources and careful dimensionality reduction are required for the large feature spaces found in genetic and neuroimaging data.

### DL challenges and limitations

SZ is a severe mental disorder and complex health issues, hence datasets are frequently limited, unbalanced, or varied. SZ presents a variety of clinical manifestations due to data heterogeneity, which makes it difficult for DL models to identify recurring patterns. Numerous sources of data, especially labeled datasets for SZ, might possess a small number of samples or imbalanced distribution of patient and non-patient groups, resulting in biased models. DL models struggle to identify specific contributing factors, such as the role of particular brain regions, biomarkers or genetic markers.

Significant computational resources are needed to train DL models using neuroimaging or multimodal data. Due to differences in demographics, genetics, and culture, models that were trained on particular populations or datasets could not function well on other populations. Unfair predictions could result from the model unintentionally learning biases if the training data is not representative.

On the other hand, DL model faces distinct challenges, primarily the need for large volumes of training data to achieve high accuracy. Transfer Learning can partially alleviate this issue by leveraging data from similar tasks, but it is not a complete substitute for primary data. Imbalanced datasets, characterized by a higher number of negative samples, are particularly problematic in biological contexts. Research indicates that training DL models on such data can yield unpredictable outcomes.

In healthcare applications, uncertainty scaling is vital for evaluating the accuracy of ML and DL diagnoses while avoiding overly optimistic predictions. Catastrophic forgetting is another challenge where new information disrupts previously learned knowledge, prompting the need to retrain models using both old and new data. Moreover, the interconnected parameters in DL models increase the risk of overfitting, leading to a divergence between the learned and actual data distributions, especially with insufficient training data. The vanishing gradient problem can also hinder DL training, particularly during backpropagation, when weight updates may fail.

**Figure 5:**
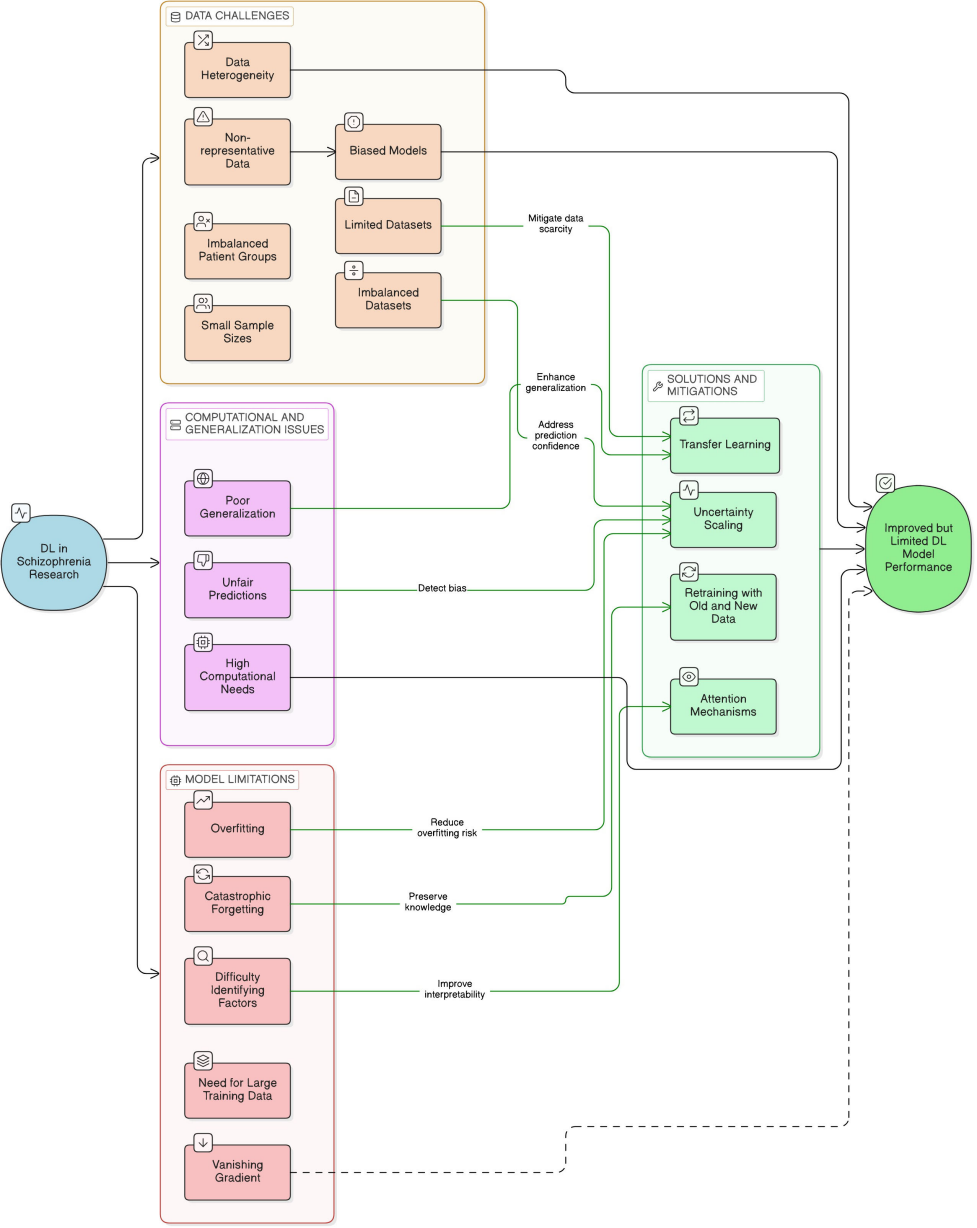
DL challenges in SZ prediction and its solutions.

The attention mechanism in DL models allows algorithms to focus on specific areas of input data during predictions. This capability can enhance SZ classification by highlighting the contributions of different brain regions and their interrelationships, aligning with the neurological aspects of cognitive processes. The overview of DL challenges and its possible responses are added in **Figure 5**.

## Conclusions

This review focuses on classifying SZ using various imaging data through AI particularly ML and DL techniques. It analyzes relevant factors from previous research and explores the potential benefits of combining EEG and neuroimaging data to enhance future diagnoses of SZ. Key findings indicate that SVM and DT are interpretable and robust, especially with smaller datasets; however, they demand more data and processing power. Our analysis covers the approaches, challenges, limitations, and results in this field.

Validity, interpretability, and ethical issues pose significant obstacles. Larger, multi-center datasets are essential to capture a diverse range of SZ cases, including early-stage symptoms and demographic variations. Notably, there is a lack of research focused on predicting clinical decompensation episodes in established SZ patients, highlighting a gap that needs to be addressed.

Integrating AI into mental health requires multidisciplinary collaboration and ethical considerations. Addressing data availability and imbalance through patient privacy solutions and synthetic data generation is crucial. The future of SZ prediction lies in the integration of multimodal data, including sMRI, fMRI, DTI, pMRI, EEG, and others. SZ symptoms emerges from psychological, biological and social factors. This multimodal integration uses the biomarkers, symptoms profile along with the functional outcomes to predict treatment response. This approach may enhance diagnostic accuracy and illuminate the complex interplay between brain function and social contexts. Hence integrating data from neuroimaging, neuropsychology, genetics and clinical task-based interviews permits for high precision care.

Advancements in DL models, such as attention mechanisms, could improve SZ classification by highlighting the significance of various brain regions. Future research should assess the practicality and effectiveness of these models in real-world clinical scenarios, ensuring their utility for healthcare professionals and patients.

## Data Availability

The original contributions presented in the study are included in the article/supplementary material. Further inquiries can be directed to the corresponding author.

## Glossary

AI: Artificial Intelligence
ALFF: Amplitude of Low Frequency Fluctuations
ANN: Artificial Neural Network
ARMS: at-risk mental state
ARMS-E: at-risk mental state early
ARMS-L: at-risk mental state late
ARMS-T: an at-risk mental state with Transition to schizophrenia
ARMS-NT: an at-risk mental state without transition to schizophrenia
BP: Bipolar Disorder
CNN: Convolutional Neural Networks
CSF: Cerebrospinal Fluid
DANS: Discriminant Autoencoder Network with Sparsity Constraint
DFA: Discriminant Function Analysis
DMN: Default Mode Network
dMRI: diffusion-weighted MRI
DL: Deep Learning
DNN: Deep Neural Networks
DT: Decision Tree
DWI: Diffusion Weighted Imaging
DTI: Diffusion Tensor Imaging
EEG: Electroencephalogram
ELM: Extreme Learning Machine
FA: Functional Anisotropy
FC: Functional Connectivity
FEP: First Episode Psychosis
FFN: Feed Forward Network
fALFF: fractional Amplitude of Low Frequency Fluctuations
GM: Grey Matter
HC: Healthy Control
ICA: Independent Component Analysis
KNN: K-Nearest Neighbors
LDA: Linear Discriminant Analysis
LSTM: Long-Short Term Memory
MD: Mean Diffusivity
MKCN: Multi-Kernel Capsule Network
MKL: Multiple Kernel Learning
MLDA: Maximum-uncertainty Linear Discriminant Analysis
ML: Machine Learning
MLP: Multilayer Perceptron
MVPA: Multivariate Pattern Analysis
PCA: Principal Component Analysis
pMRI: perfusion MRI
ReHo: Regional Homogeneity
RA: Relative Anisotropy
ROI: Region of Interest
RF: Random Forest
RNN: Recurrent Neural Network
sMRI: structural MRI
SNP: Single Nucleotide Polymorphism
SVM: Support Vector Machine
SVR: Support Vector Regression
SZ: Schizophrenia
TL: Temporal Lobe
WM: White Matter
